# Acute dacryocystitis retention: a case report and literature review

**DOI:** 10.5935/0004-2749.20220044

**Published:** 2022

**Authors:** Shaikha Aleid, Silvana A. Schellini, Osama Alsheikh, Sahar M. Elkhamary

**Affiliations:** 1 Oculoplastics Department, King Khaled Eye Specialist Hospital, Riyadh, Saudi Arabia; 2 Department of Ophthalmology, Faculdade de Medicina, Universidade Estadual de São Paulo “Júlio de Mesquita Filho”, Botucatu, SP, Brazil; 3 Radiology Department, King Khaled Eye Specialist Hospital, Riyadh, Saudi Arabia

**Keywords:** Dacryocystitis, Lacrimal duct obstruction, Lacrimal apparatus diseases, Nasolacrimal duct, Dacriocistite, Obstrução dos ductos lacrimais, Doenças do aparelho lacrimal, Ducto nasolacrimal

## Abstract

Acute dacryocystitis retention (ADR) is an unusual entity that contributes to an incorrect diagnosis and treatment. We describe a case of acute dacryocystitis retention occurring in a 61-year-old diabetic male who presented with severe pain, swelling, and inflammatory signs above the left medial canthal ligament tendon. He had no previous history of epiphora. Computed tomography scan indicated acute dacryocystitis. Clinical treatment resulted in complete resolution of the condition. Syringing one month after the acute episode indicated a patent lacrimal excretory system. The temporary obstruction that evolved to an acute dacryocystitis retention was probably secondary to nasal alteration or supposed dacryoliths. Timely, conservative clinical treatment can lead to complete resolution of acute dacryocystitis retention with no further treatments.

## Abstract (pt)

A dacriocistite aguda de retenção é uma entidade incomum, o que contribui para que o diagnóstico e o tratamento não sejam corretos. Estamos descrevendo um caso de dacriocistite aguda de retenção ocorrendo em um homem diabético de 61 anos que apresentou dor intensa, edema e sinais inflamatórios acima do tendão cantal medial esquerdo. Ele não tinha histórico anterior de epífora. A tomografia computadorizada indicou dacriocistite aguda. O tratamento clínico resultou na resolução completa da condição. A irrigação, um mês após o episódio agudo, indicou sistema excretor lacrimal pérvio. A obstrução temporária que evoluiu para uma dacriocistite aguda de retenção foi provavelmente secundária a alteração nasal ou supostos dacriolitos. O tratamento clínico conservador pode levar à resolução completa da dacriocistite aguda de retenção, sem necessidade de outros tratamentos.

## INTRODUCTION

Acute dacryocystitis is characterized by pain, previous epiphora, erythema, and swelling generally located below the medial canthal ligament tendon^(1,2)^. A relatively new condition, called acute dacryocystitis retention (ADR) or reversible obstruction, has not been well described and less often recognized^(3-5)^. Patients with ADR presented with a resolution of the condition after spontaneous expulsion of casts to the nose or mouth^(3)^. Subsequently, differences between ADR and classical acute dacryocystitis were highlighted, as the rapid and sudden onset of severe pain and tearing with minimal but tender distention of the lacrimal sac and temporary or permanent, partial or total obstruction, suggesting the term noninfectious ADR^(4-6)^.ADR represents 0.8 cases per year in a subspecialty oculoplastic practice and can be present in 23.5% of patients with acquired nasolacrimal duct obstruction (NLDO) together with dacryoliths^(7)^.

Due to some overlap in symptoms at presentation, there is a risk that ADR may be misdiagnosed as simple acute dacryocystitis. Thus, it is important to document cases of ADR to improve the diagnosis as the treatment is diverse. We present an even more unusual case of ADR with lacrimal sac enlargement above the medial canthal ligament tendon and its management. The Ethical Committee Research Board of King Khaled Eye Specialist Hospital approved this report.

## CASE REPORT

A 61-year-old male presented to our hospital in 2018, complaining of severe pain and progressive swelling in the medial aspect of the left upper eyelid 3 days prior to presentation. The patient denied previous tearing or other symptoms, trauma, or nasal surgery. He had controlled diabetes and was otherwise healthy.

**Figure 1:**
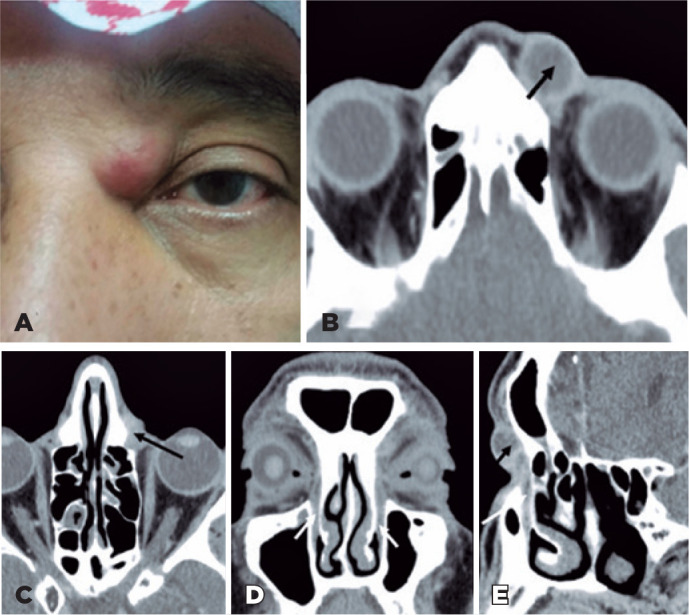
(A) Clinical photo showing left inner canthus swelling with inflammatory signs located above the medial canthal ligament tendon. (B-C) Axial post-contrast CT scan images revealed a well-defined, ovoid-shaped marginally enhanced lesion of near-fluid density noted anteriorly to the orbital septum and above the left medial canthus (black arrow). (D) Coronal and (E) sagittal reconstruction from the CT scan image showing a mucous fluid-filled normal-sized nasolacrimal sac (white arrow) with a normal diameter of the intraosseous funnel-shaped nasolacrimal ductus extending from the lacrimal sac and ending in the inferior nasal meatus, below the inferior turbinate (long white arrow). Hypertrophic inferior nasal turbinate and deviated bony nasal septum toward the affected side. No differences were observed between the right or left nasolacrimal sac or nasolacrimal duct width, length, or angulation slope. No evidence of deep orbital inflammation.

On examination, a firm painful mild erythematous tender mass was noted above the medial canthal tendon on the left side (Figure 1A). No abnormalities of the lacrimal puncta or spontaneous reflux were noted. The ocular exam was unremarkable bilaterally.

Computed tomography scan (CT scan) revealed the measurement of the left lacrimal sac as 1.6 cm × 1.3 cm × 2.3 cm and identified it as a well-defined marginally enhanced hypodense ovoid-shaped sac, filled with fluid-mucous secretion, enlarged just above the medial canthal ligament tendon, suggestive of acute dacryocystitis. The inflammatory reaction did not extend to the orbit. There was no dacryolith inside the lacrimal system. Coronal CT reformations of the axial images showed the entire course of the nasolacrimal ductus with funnel-shaped terminus juxta-lateral to the inferior nasal turbinate that was hypertrophic with a deviated bony nasal septum toward the left side (Figure 1B-E). Clinical treatment was started with 1 Gr oral amoxicillin and clavulanate potassium (Augmentin, SmithKline Beecham Ltd., Worthing, UK) and topical ophthalmic erythromycin ointment (erythromycin, API, Amman, Jordan) twice daily. One month later, the patient presented with a resolution of the acute dacryocystitis. The dye disappearance test was normal, regurgitation test was negative, and lacrimal syringing test indicated patency, suggesting an ADR diagnosis. Two years later, the patient was doing fine and had no more signs of inflammation in the lacrimal system.

## DISCUSSION

This case reports a well-documented ADR associated with the enlargement of an inflamed lacrimal sac located above the medial canthal ligament tendon. Distention of the lacrimal sac in cases of dacryocystitis is usually located below the medial canthal ligament tendon because the lower portion of the lacrimal sac is covered only by the capsule-palpebral fascia^(8)^. However, our case presented with enlargement of the lacrimal sac above the medial canthal ligament tendon, which is seldom observed.

The ADR as well as the idiopathic NLDO usually affects females under 40 years of age^(7)^. However, we report an older male with no previous history of epiphora who suddenly developed pain and inflammation in the medial canthal tendon area. Prodromal epiphora is uncommon in cases of ADR^(4)^. The low humidity and desert conditions in Saudi Arabia can also contribute to a lack of tearing.

The severe pain associated with ADR is likely due to a sudden acute mechanical blockage of the lacrimal outflow with rapid distention of a sac that was not previously dilated as in chronic dacryocystitis, provoking intense pain^(2,6)^.

The pain prevented diagnostic tests as regurgitation on pressure or syringing at the first visit, as reported^(4)^. However, one month later, the dye disappearance test was normal, the regurgitation test was negative, and the syringing test showed patency of the lacrimal excretory system, proving the absence of obstruction after the acute episode in our patient.

Syringing of the lacrimal passage is a simple exam, with a high level of confidence to detect patency or lacrimal obstruction. However, anatomical image exams such as dacryocystography or CT scan are considered a safe and time-efficient method to assess the lacrimal system in patients with epiphora and are indicated to confirm diagnosis^(9)^.

CT scan was performed in our patient without contrast in the lacrimal system because the painful process at presentation prevented catheterization of the lacrimal system. However, it was possible to clearly observe the signs of acute dacryocystitis, a well-distinct image of the lacrimal sac enlarged just above the left medial canthal ligament tendon and toward in continuation with the nasolacrimal duct, with no dacryoliths.

Although CT scan is not routine to evaluate watery eyes, it provides excellent contrast resolution between bony structures and surrounding soft tissues, making it possible to observe the nasolacrimal duct within the bone channel in a funnel shape, soft tissue opacities (full opacity, partial opacity, or no opacity) reflecting air inside the lacrimal system, mucosal edema/thickening within the lacrimal system, and retention of secretions^(10)^. Other drainage-limiting factors such as bony abnormalities, obstructive masses, and nearby anatomical structures are readily identified on CT scan, and observing these nasal alterations in our patient was decisive, such as hypertrophy of the inferior nasal turbinate with a deviated bony nasal septum toward the affected side, increasing the suspicion of nose alterations as the cause of temporary obstruction of the lacrimal system in our case.

CT scan in our case was also an important tool for ruling out other differential diagnoses such as dermoid cyst, encephalocele, or frontal sinus mucocele that usually presents in the upper internal quadrant of the orbit, superior to the medial canthal ligament tendon.

Management with antibiotics resulted in good outcomes in this reported case. However, the evolution of ADR does not depend on antibiotic therapy since there is only temporary obstruction of the passage of tears.

The reversible cause of ADR in our case can be related to anatomical nasal variations or even partial nasal obstruction, as previously reported^(6)^. Temporary or permanent ADR can be secondary to lacrimal sac diverticula, vascular engorgements, specific chronic inflammation (rhinosporidiosis, Epstein-Barr virus), lymphoid hyperplasia^(5)^, or mucous plug, small dacryoliths, blood clot, or other foreign bodies suddenly impacting and occluding the nasolacrimal duct and then spontaneously expelled^(4,6)^.

In cases of persistent obstruction, syringing or probing of the lacrimal outflow system can be performed. External or endoscopic dacryocystorhinostomy with or without inferior turbinate fracturing should be considered if recurrence of acute episodes occurs^(5,7)^.

In conclusion, we report an older diabetic male with no previous epiphora who developed an ADR, manifested by severe pain and inflammation distending the lacrimal sac above the medial canthal ligament tendon. Clinical and imaging investigations revealed a temporary obstruction probably secondary to nasal alteration or supposed dacryoliths. Timely, conservative clinical treatment resulted in complete resolution of the condition.
